# Ultralow power consumption gas sensor based on a self-heated nanojunction of SnO_2_ nanowires†

**DOI:** 10.1039/c8ra06061d

**Published:** 2018-10-25

**Authors:** Trinh Minh Ngoc, Nguyen Van Duy, Chu Manh Hung, Nguyen Duc Hoa, Nguyen Ngoc Trung, Hugo Nguyen, Nguyen Van Hieu

**Affiliations:** International Training Institute for Materials Science (ITIMS), Hanoi University of Science and Technology Hanoi Vietnam nguyenvanduy@itims.edu.vn; School of Engineering Physics, Hanoi University of Science and Technology Hanoi Vietnam; Uppsala University, Department of Engineering Sciences Lägerhyddsvägen 1 751 21 Uppsala Sweden; Faculty of Electrical and Electronic Engineering, Thanh Tay Institute for Advanced Study (TIAS), Thanh Tay University Yen Nghia, Ha-Dong District Hanoi 10000 Vietnam; Phenikaa Research and Technology Institute (PRATI), A&A Green Phoenix Group 167 Hoang Ngan Hanoi 10000 Vietnam

## Abstract

The long duration of a working device with a limited battery capacity requires gas sensors with low power consumption. A self-heated gas sensor is a highly promising candidate to satisfy this requirement. In this study, two gas sensors with sparse and dense SnO_2_ nanowire (NW) networks were investigated under the Joule heating effect at the nanojunction. Results showed that the local heating nanojunction was effective for NO_2_ sensing but generally not for reduction gases. At 1 μW, the sparse NW sensor showed a good sensing performance to the NO_2_ gas. The dense SnO_2_ NW network required a high-power supply for gas-sensitive activation, but was suitable for reduction gases. A power of approximately 500 μW was also needed for a fast recovery time. Notably, the dense NW sensor can response to ethanol and H_2_S gases. Results also showed that the self-heated sensors were simple in design and reproducible in terms of the fabrication process.

## Abstract 2

We realize the local self-heated nanojunction in nanowires for ultralow power consumption gas sensor by a simple design and fabrication process.

## Graphical abstract

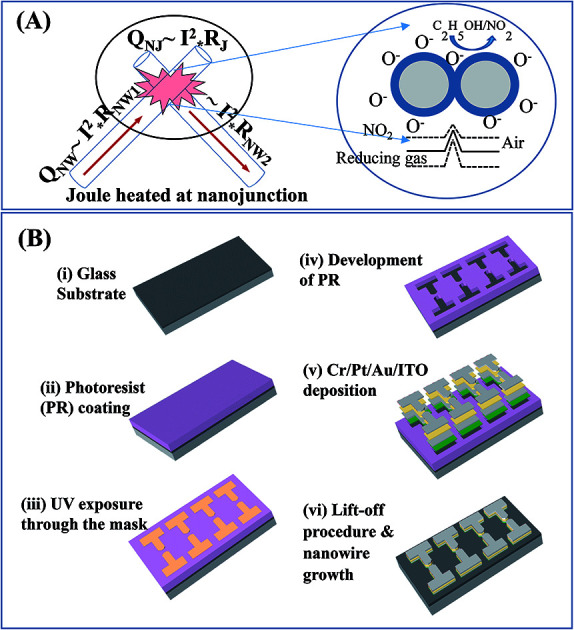

## 1. Introduction

In recent years, studies on semiconducting metal oxide (SMO) gas sensors have focused primarily on one- and two-dimensional nanostructured materials due to their advantages of large surface area and high porosity that favour adsorption and thus detection of gas molecules.^1–4^ These high-crystalline structures are superior to amorphous materials and nanoparticles in terms of sensor durability and stability.^4,5^ When catalytic materials are introduced onto the surface of these one- and two-dimensional nanostructures, their gas-sensitive properties can be enhanced.^6–8^ Many methods of fabrication and integration of materials onto the sensor have been developed, including drop-coating, spray-coating, spin-coating, printing and direct growth on the electrode. Amongst them, the SnO_2_ nanowires (NWs) directly grown on-chip have been reported as the most effective method with high application potential due to their controllable and reproducible fabrication process and the strong and reliable formation of the material layer.^3,9,10^ However, development of a small, lightweight, high-performance, low-cost and ultralow-power-consumption gas sensor for integration into wireless and portable devices is challenging in the era of the Internet of Things.^11–13^ Conventional SMO gas sensors generally require heat to activate the gas-sensing process. The heater, often integrated on-chip, consumes significant electrical power and complicates the device design and fabrication.^11,12^ To minimize the size and power consumption, the Japanese company Figaro has recently succeeded in producing commercial MEMS-based sensors with considerably small dimensions of 0.99 mm × 2.5 mm × 3.2 mm and a power consumption of approximately 15 mW.^11^ However, the MEMS-based designs of gas sensors still display drawbacks, such as long and complicated production processes, and poor adhesion of the on-chip heater and the gas-sensitive material layer on the substrate surface.^12^

In recent years, some research groups have successfully fabricated gas sensors with self-heated metal oxide NWs,^13–18^ which require no heater structure. In this case, the metal oxide NWs constitute both the heater and the gas-sensing material. The Joule self-heating effect reduces the manufacturing cost due to their simple designs and fabrication processes. The power consumption of self-heated sensors is generally lower than those of sensors that use conventional on-chip or external heater.^18^ Gas sensors that use the self-heating effects of carbon nanotubes and nanofibers^19,20^ and single-stranded NW^14,15,21,22^ have been studied. Recently, the self-heating effects on a core–shell structure have also been investigated.^16^ Results showed that the sensors can operate at a substantially low power (in the order of μW). Nonetheless, the core–shell structure sensors, despite their low power consumption, are difficult to fabricate and still suffer from low reproducibility. In 2011, Lian Feng Zhu *et al.*^17^ studied a Pt-coated W_18_O_49_ NW network sensor with a simple design and fabrication process. The sensor shows good sensitivity and selectivity to H_2_, but the power consumption remains high at 30–60 mW. In our previous research, the SnO_2_ NW network works as a self-activated sensor. However, the power consumption can be reduced only to 20–30 mW when used for NO_2_ gas detection due to the non-optimised structure.^18^ An ultralow-power and self-heated sensor developed using a simple fabrication process is necessary for battery-free and mobile-sensing devices. At a consumed power level of 10 μW, the self-heated sensor can easily function with an energy harvesting source. However, the μW power consumption of the self-heated sensor can be obtained only in a single NW^15^ or in functionalised NW devices.^16^ Optimising the sensor structure to decrease the power consumption of a device with sufficient sensing performance is still a difficult task.

In the present study, we aim to develop effective self-heated SnO_2_ NW network sensor to further reduce power consumption by controlling the junction density and the NW network area. We also discuss the gas-sensing mechanism of the devices. In addition, for the first time we study about the damage by powering in the self-heated NW sensors.

## 2. Experimental

**Fig. 1:**
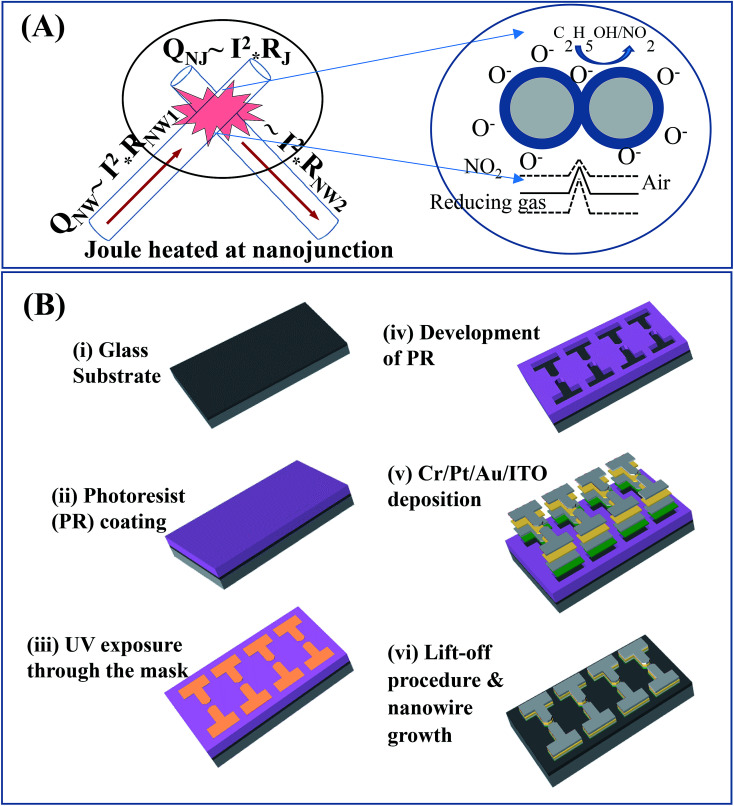
(A) Design of nanojunction-heated gas sensor and (B) fabrication processes.

The design and fabrication of nanojunction-heated NW sensors are illustrated in Fig. 1. The design of sensor involves the SnO_2_ NWs grown on a glass substrate. Parameters of the growth process were controlled to obtain the desired length of NW and the density of nanojunctions between electrodes (Fig. 1(A)). The fabrication process of the sensor is described by the following steps: platinum electrodes were patterned on a heat-resistant glass substrate with the size of 15 mm × 10 mm and thickness of 500 μm, as shown in Fig. 1(B). The gap between the two opposite electrodes was 2 μm. The tin oxide NWs were grown using thermal chemical vapour deposition (CVD) method.

A quartz boat containing approximately 0.1 g of tin (purity of 99.9%) was placed at the centre of a quartz tube horizontal furnace. The entire system was firstly purged with Ar with a flow rate of 300 sccm for 5 min. The heat was increased at a rate of 36 °C min^−1^. The CVD process was subsequently carried out at the temperature of 715 °C, with an O_2_ gas flow of 0.5 sccm at the pressure of 1.8 × 10^−1^ torr. To obtain different densities of the SnO_2_ NWs, the growth time was set to 10 and 20 min (for sensors S1 and S2, respectively). After the CVD growth step, the furnace was naturally cooled down to room temperature. Afterwards, the sensors were removed to examine gas-sensing properties. Details on the gas sensing measurement system are described elsewhere.^18^ The sensor response was determined as the ratio of transient resistance to resistance in the dry air (denoted as *R*_air_) at the same working temperature. The electric supply power was maintained at different constant levels during measurement to hold the sensor material at different constant working temperatures.

## 3. Results and discussion

**Fig. 2:**
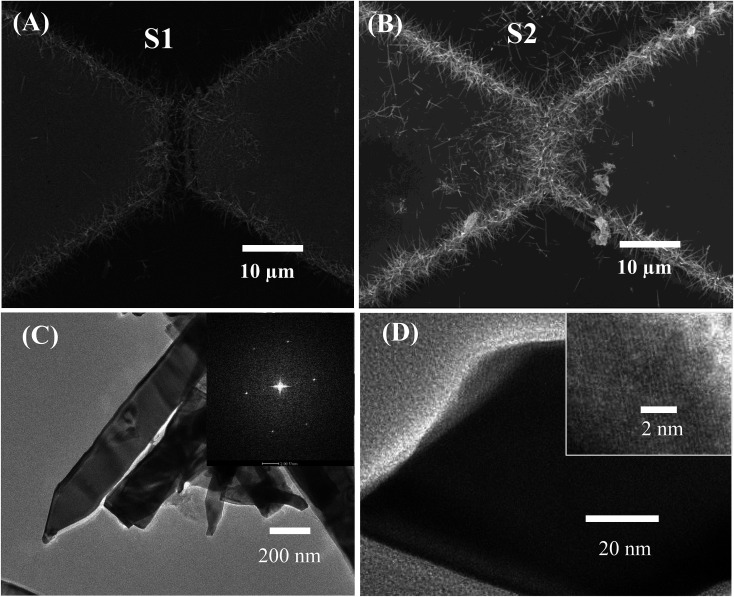
SEM images of (A) sparse nanowires (S1), (B) dense nanowires (S2), and (C and D) TEM images of SnO_2_ nanowires. Inset of (C) is the SAED; inset of (D) is the HRTEM image.

The morphology of the SnO_2_ NW networks was characterised by field emission scanning electron microscopy (FESEM), as shown in Fig. 2. The SnO_2_ NWs show no growth on the entire glass surface but only from the edge of the electrodes bridging the gap between them. Thus, the electric-conducting channel of the sensor is composed of the NWs and their junctions. As shown in Fig. 2(A), sensor S1 presents a highly sparse NW network so that the gap between the electrodes can still be observed. By contrast, sensor S2 (Fig. 2(B)) possesses a considerably dense NW network that obscures the picture of the gap. Accordingly, S2 may be able to accumulate a large amount of heat so that the network can reach a higher working temperature than that of S1. However, S2 may consume higher power than S1, which will be discussed later. TEM images of the synthesised SnO_2_ NWs are shown in Fig. 2(C and D), wherein the lattice fringes are clearly observed (inset of Fig. 1(D)). The SAED of the NW shown in the inset of Fig. 2(C) displays bright spots, and this phenomenon confirms the single crystallinity of the material.

**Fig. 3:**
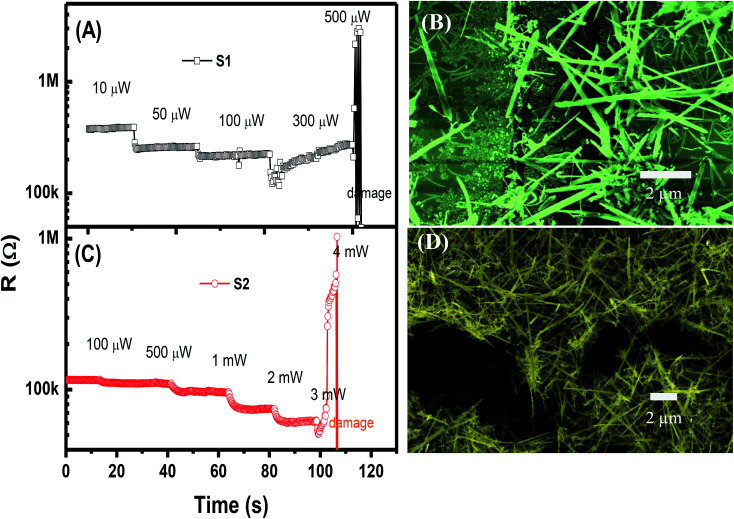
Time dependency of sensor resistance under different applied powers, and corresponding SEM images of the devices after damage: (A and B) sensor S1; (C and D) sensor S2.

In the self-heated sensor, the sensing element (NWs) is also the heater. The temperature of the sensor can be controlled by applying an external voltage (current) for signal measurement. In our study, the different applied powers to the devices range from 1 μW to 3 mW. The high supplied power can damage the device. Thus, we studied the change in resistance of the sensors under various supplied powers in dry air, as shown in Fig. 3(A and C). For sensor S1, when the power ranging from 1 μW to 300 μW is applied, the resistance decreases; this condition confirms the self-heated effect. However, at a supplied power of 500 μW, the sensor is damaged as documented by the sudden increase in resistance. Low-magnification SEM image of S2 after damage shows the electrodes with the discontinuity of NWs (ESI Fig. S3†). The SEM image of S1 after measurement with a supplied power of 500 μW shown in Fig. 3(B) confirms the damage of the device. On the contrary, S2 exhibits no change in sensor resistance when a power lower than 100 μW is applied. As the power increases from 100 μW to 3 mW, the sensor resistance decreases considerably as a result of the increase in temperature. S2 is damaged at a supplied power of 4 mW. The SEM image of the damaged S2 is shown in Fig. 3(D).

**Fig. 4:**
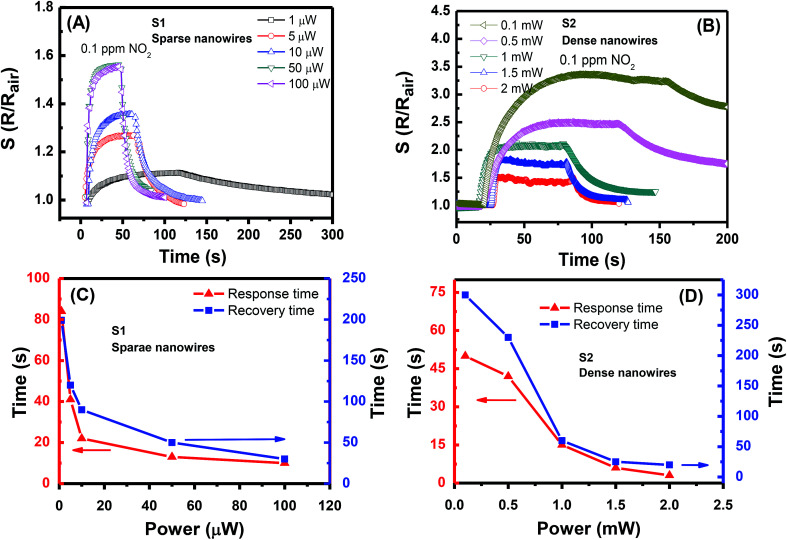
Transient gas responses of S1 and S2 (A and B, respectively) to 0.1 ppm NO_2_ at different supplied power values and their response/recovery times (C and D, respectively).

The power range supplied for each sensor was determined for fast sensing performance, *i.e.*, short response/recovery times. Hence, only the sensing results with response/recovery times below 300 s will be presented and discussed. The transient responses of S1 and S2 to 0.1 ppm NO_2_ gas are presented in Fig. 4(A and B), respectively. Evidently, both sensors show an increase in resistance upon exposure to NO_2_ gas. The reason is that the NO_2_ molecules adsorb on the surface of SnO_2_ and capture free electrons to form the depletion region because the SnO_2_ is an n-type semiconductor with electron as the main carrier; this phenomenon results in the increase in sensor resistance.^10,18^ The S1 with sparse NW network requires only a low power for an excellent sensing performance. At a supplied power of 1 μW, S1 shows a response of 1.1–0.1 ppm NO_2_ gas, which is the lowest response in the measurement series. This value is the lowest power consumption recorded to date for gas sensor. The lowest value prior to this record is 20 μW for the self-heated single SnO_2_ NW sensor presented in.^15^ The gas response increases with the increase in supplied power. The highest gas response of 1.7 is observed at the working power of 50 μW. Further increase in the power to 100 μW causes no improvement in the gas response but slightly decreases it. To estimate the working temperature of S1 at different supplied powers, the transient resistance *versus* time upon exposure to 1 ppm NO_2_ at the temperature ranging from 150 °C to 450 °C was tested. The results are shown in ESI Fig. S1(A).† The sensor response increases with the increase in the working temperature from 150 °C to 300 °C and decreases with the further increase in working temperature (Fig. S1(B)).† The sensor responses are higher than the values obtained by applying different powers. However, the increasing trend of sensor response with the increase in supplied power is consistent with the increase in response *versus* temperature ranging from 150 °C to 300 °C.

The supplied power of S2 is in the range of 0.1–2 mW. Thus, the lowest supplied power is two orders higher than that of S1, and the highest supplied power is 40 times higher ditto. Notably, at less than 0.1 mW, the gas response increases with the increased supplied power; this trend is similar to that of S1, but the recovery time is out of the concerned limit. Therefore, those results are excluded here. Within the supplied power range, the gas response of S2 decreases with the increased supplied power. The highest response to 0.1 ppm NO_2_ is approximately 3.5 at 0.1 mW, and the lowest value of 1.5 is observed at 2 mW. When considering the response/recovery times, Fig. 4(C and D) show that the response speed of both sensors is faster than their recovery speed. Generally, the sensing performance speed is enhanced with the increased working power. To obtain both a high gas response and reasonable performance speed for S1, the working power should be 10 μW with response/recovery times of 22/90 s. The preferred working power for S2 is 1 mW with response/recovery times of 15/60 s. According to the obtained results, a certain level of heat is required so that the NO_2_ gas will be activated. Without heating, the individual NW junction in S1 cannot effectively absorb the gas molecules that cause the sensor resistance change. The optimal working state of the gas sensor is reached at the balance of the gas absorption and desorption processes. Beyond this state, *i.e.*, at a high temperature, the gas response decreases when the desorption process dominates. The NW network of S2 is considerably denser than that of S1, thereby resulting in long response/recovery times. At the supplied power of 0.5 mW, S2 requires more than 200 s to resume the previous resistance value. Evidently, S1 with sparse SnO_2_ NW network is a NO_2_ gas sensor with ultralow power consumption. Nevertheless, S2 remains a good NO_2_ gas sensor that requires a low power; this sensor is also a robust sensor due to its dense NW network.

**Fig. 5:**
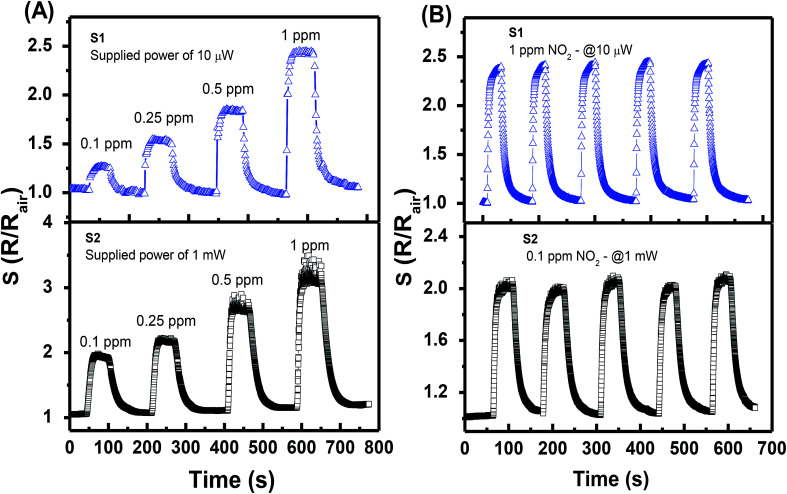
Transient responses of S1 and S2 to different NO_2_ gas concentrations (A), and the repeatability test of five response cycles (B).

The transient response characteristics of sensors S1 and S2 to NO_2_ gas were investigated (Fig. 5(A)) at their preferable supplied power, *i.e.*, 10 μW and 1 mW, respectively, for comparison. At the NO_2_ gas concentration of 0.1–1 ppm, S1 exhibits slightly lower responses than those of S2, *i.e.*, 1.27–2.45 *vs.* 1.95–3.2. Despite that results also show that both sensors can easily distinguish a change in NO_2_ gas concentration with the step of 0.15 ppm. Sensor S1 can monitor NO_2_ gas at a very low power of 1 μW with excellent sensing performance (ESI Fig. S2†). Repeatability tests with five response cycles in a row (Fig. 5(B)) were also carried out for both sensors. The two sensors display a negligible variation (less than 5%) in response values. The results suggest that the power consumption of the self-heated sensor can be decreased one or two orders with the response in the same order by controlling the density of nanojunction. The long-term stability of the sensor was tested over 10 cycles after a month of continuous operation at a supplied power of 10 μW, and the data are shown in ESI Fig. S4.† The results show no considerable distortion of sensing performance, which indicates the excellent stability of the device.

**Fig. 6:**
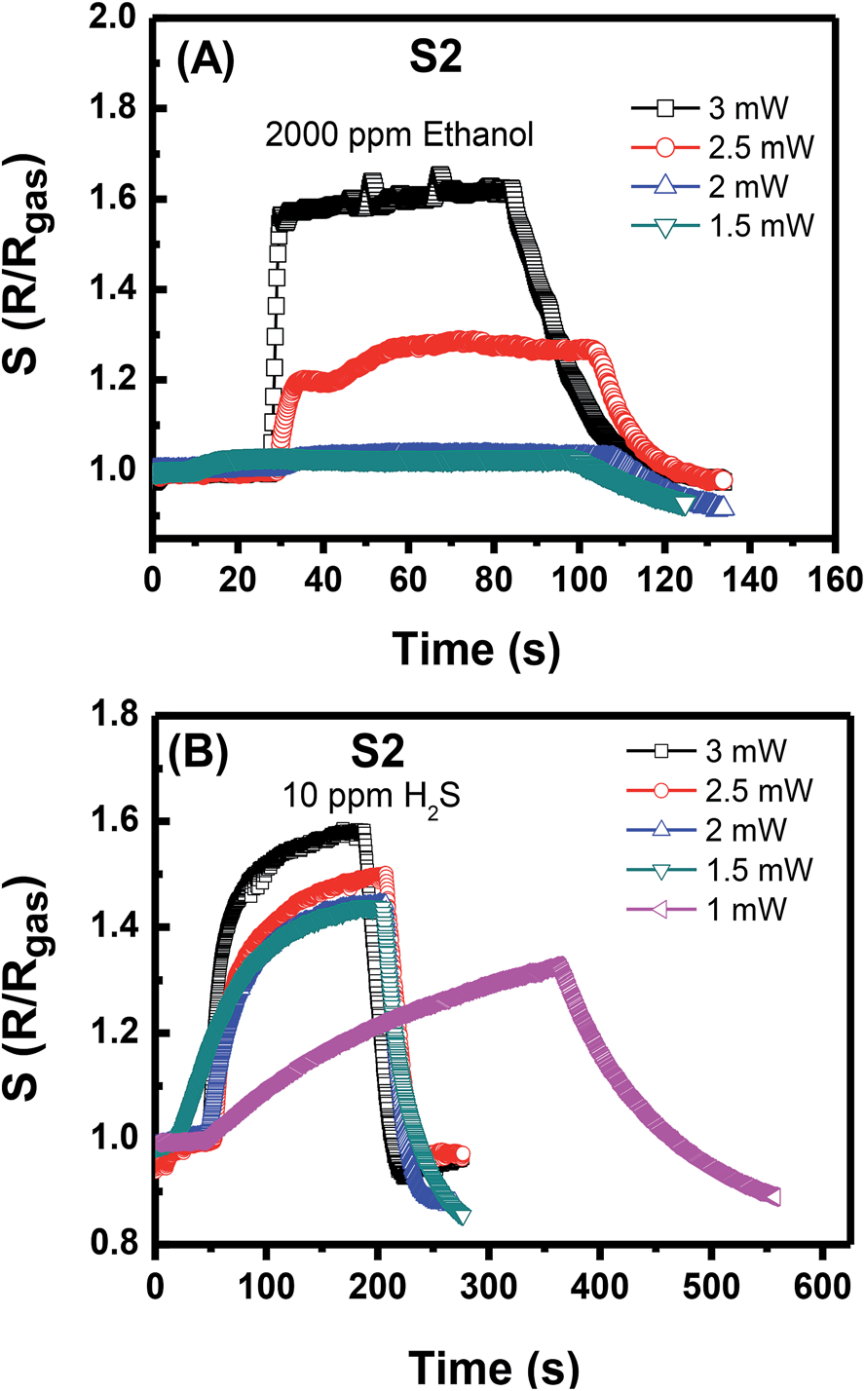
Response to (A) ethanol and (B) H_2_S of sensor S2 at different powers.

All these performance characteristics demonstrate that both sensors are suitable for practical application in monitoring highly toxic NO_2_ at low concentrations. The excellent sensing properties and gas selectivity are the advantages of the self-heated sensors. The activation energy for gas absorption and reaction can be modified by controlling the supplied power corresponding to the heat. The response to reduction gases, such as ethanol and hydrogen sulfide, was also examined. S1 shows no significant resistance change for 2000 ppm of ethanol at the supplied power of 100 μW (data not shown). S2 shows the ability to work at a high supplied power that can reach 3 mW. Fig. 6(A) shows that at supplied power of 2.5 mW, S2 can relatively respond rapidly to 2000 ppm of ethanol with the *R*/*R*_gas_ ratio of approximately 1.25. At 3 mW, the ethanol response value reaches 1.6. Notably, the supplied power was not increased to a value higher than 3 mW to avoid damage. At 10 ppm of H_2_S gas (Fig. 6(B)), the S2 starts to work at the low power of 1 mW with a long response time of more than 400 s. At the supplied power that is higher than 1.5 mW, the gas response and recovery times are considerably improved. Therefore, the dense NW network can work at a higher temperature than that of the sparse one. This deduction is supported by the discussion of O. Monereo *et al.* in,^23^ wherein they suggested that most of the power will be dissipated in the most resistive region to reach the high temperature for the resistors in a series. When the contact resistance decreases due to the increase in the number of contact nodes (as in the case of S2 sensor), the heat will distribute mostly on the NW segments. Consequently, S2 but not S1 can be used for sensing of reduction gases. This utilisation is possibly limited by the low working power of S1, which is insufficient to reach the activation temperature for reduction gas to react with the sensing material due to the heat loss to the environment. Dense NW heating was investigated in our previous work with thermal emission microscopic images.^18^ In the present study, the sensor heating area is remarkably reduced compared with that in the previous one.

**Fig. 7:**
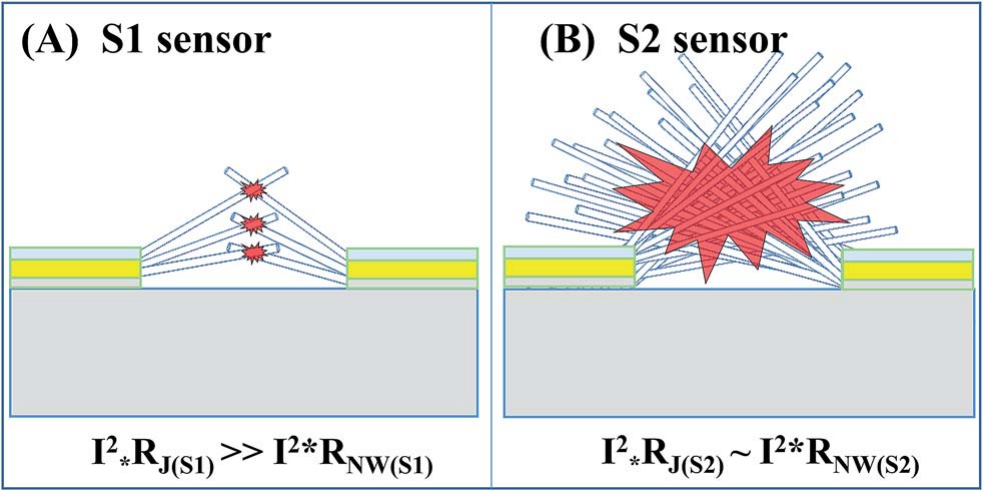
Sensing mechanisms of nanojunction-heated sensors: illustration of nanojunction local heating (A) and bundle heating (B).

To explain the obtained results in detail, the local nanojunction heating model for S1 and the bundle heating model for S2 were suggested. The local nanojunction heating for the sparse NWs in S1 is illustrated in Fig. 7(A). The nanojunction between two NWs has a higher resistance (*R*_j_) than that of the NW (*R*_NW_). Therefore, when the device is powered with an electric current (*I*), the Joule heating of the nanojunction (*Q*_NJ_), proportional to *I*^2^ × *R*, is higher than that of the NW (*Q*_NW_). The highly separated and very small junctions in S1 are considered local heat sources that cannot increase the total NW network temperature. Nevertheless, the large number of junction nodes in S2 decreases the junction resistance considerably. In other words, junction and NW body contribute to the heating. The dense NW sensor will be heated by the entire NW bundle due to the collective heating (as shown in Fig. 7(B)). S1 and S2 can respond to NO_2_ gas due to the low activated temperature of this gas of approximately 100 °C.^24–27^ However, a difference in response/recovery time is observed between the two sensors due to the dissimilar gas diffusion speeds. In general, the working temperature of SMO material for ethanol gas is nearly 200 °C or above^28,29^ and lower for H_2_S gas.^30,31^ Thus, the reduction gas detection of S2 depends on its bundle heating effect, wherein high supplied power generates high temperature. In summary, the sparse NWs and local heating at nanojunctions on S1 enable the ultralow power consumption of the NO_2_ sensor, whereas S2 with its networked heating in dense NWs consumes high power but is effective for reducing gases.

## 4. Conclusions

Two self-heated gas sensors based on SnO_2_ NW networks with a simple design and fabrication process are developed. The local heating phenomenon of NW junctions explains the ultralow power consumption of the sensors. Both the sparse and dense NW networks demonstrate their abilities to detect NO_2_ with the concentration of 0.1 ppm at the supplied power that is less than 100 μW. Nevertheless, the response/recovery times of the sparse NW sensors are remarkably reduced at the preferable operating power of 10 μW. Results show that the local heating of the sparse NW junctions is ineffective for sensing reduction gases but the collective heating of the dense NWs is. Finally, the self-heated SnO_2_ NW network is presumed to stipulate the concept of a new-generation gas sensor based on SMO materials with ultralow power consumption in the future.

## Conflicts of interest

There are no conflicts to declare.

## Supplementary Material

RA-008-C8RA06061D-s001
