# Supplementary material for: Ultralow power consumption gas sensor based on a self-heated nanojunction of SnO2 nanowires
Source: RSC Adv. 2018 Oct 25;8(63):36323–30. doi: 10.1039/c8ra06061d (PMC9089278; doi:10.1039/c8ra06061d)

Electronic Supplementary Material (ESI) for RSC Advances.
This journal is © The Royal Society of Chemistry 2018

Electronic Supporting Information

Ultralow Power Consumption gas Sensor based on Self-heated Nanojunction of SnO_2 Nanowires

Trinh Minh Ngoc[1], Nguyen Van Duy[1,*], Hugo Nguyen[2], Chu Manh Hung[1], Nguyen Ngoc Trung[3], Nguyen Duc Hoa[1], Nguyen Van Hieu[4,5]

[1)]International Training Institute for Materials Science, Hanoi University of Science and Technology, Hanoi, Viet Nam

[2)]School of Engineering Physics, Hanoi University of Science and Technology, Hanoi, Viet Nam

[3)]Uppsala University, Department of Engineering Sciences, Lägerhyddsvägen 1, 751 21 Uppsala, Sweden

[4)]Faculty of Electrical and Electronic Engineering, Thanh Tay Institute for Advanced Study (TIAS), Thanh Tay University, Yen Nghia, Ha-Dong district, Hanoi 10000, Viet Nam

[5)]Phenikaa Research and Technology Institute (PRATI), A&A Green Phoenix Group, 167 Hoang Ngan, Hanoi 10000, Viet Nam

*E-mail: nguyenvanduy@itims.edu.vn

Figure S1. (A) Transient resistance vs. time upon exposure to 1 ppm NO_2 measured at different temperatures of sensor S1; (B) sensor response as a function of working temperatures.

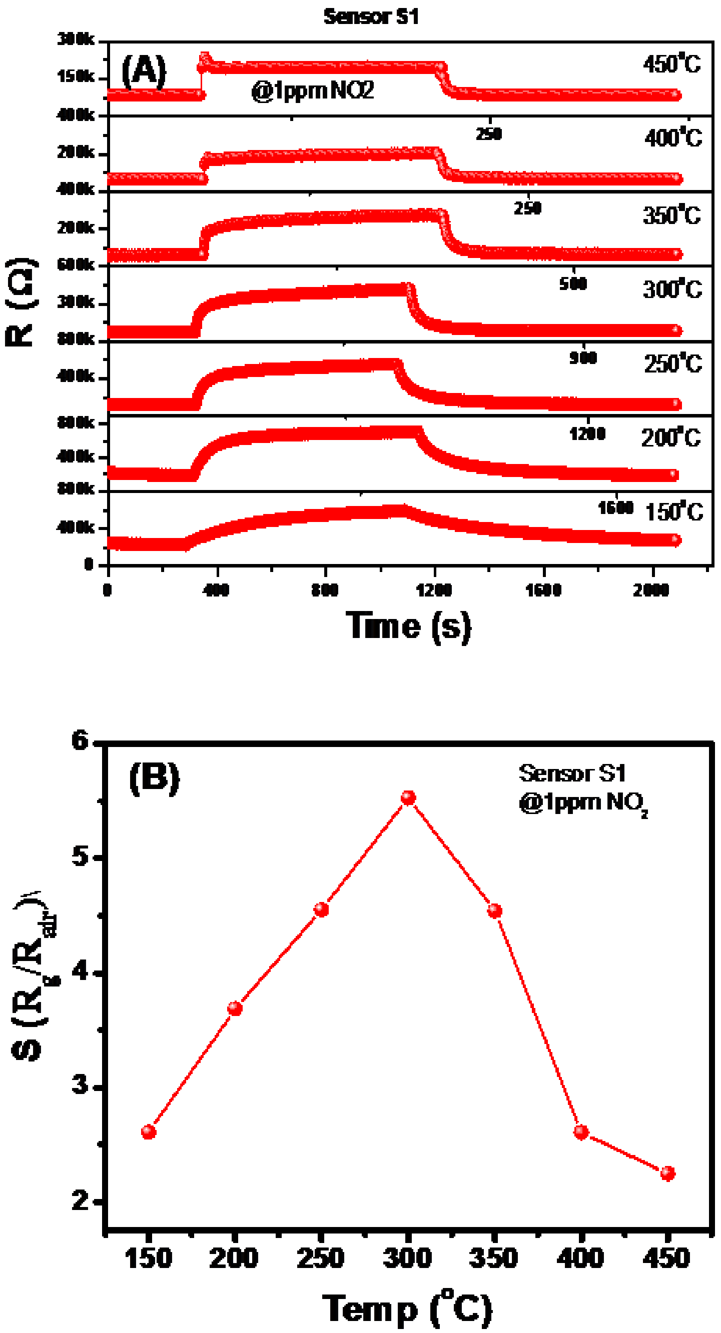

Figure S2. Transient resistance vs. time upon exposure to various NO_2 concentrations measured at different powers of sensor S1.

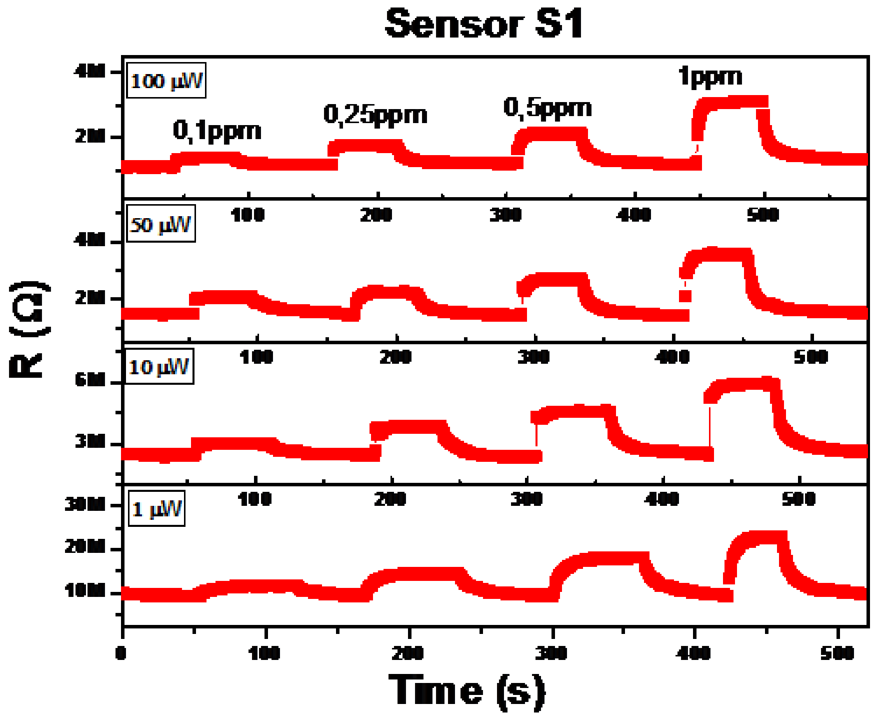

Figure S3. Low magnification SEM image of sensor S2 after damage.

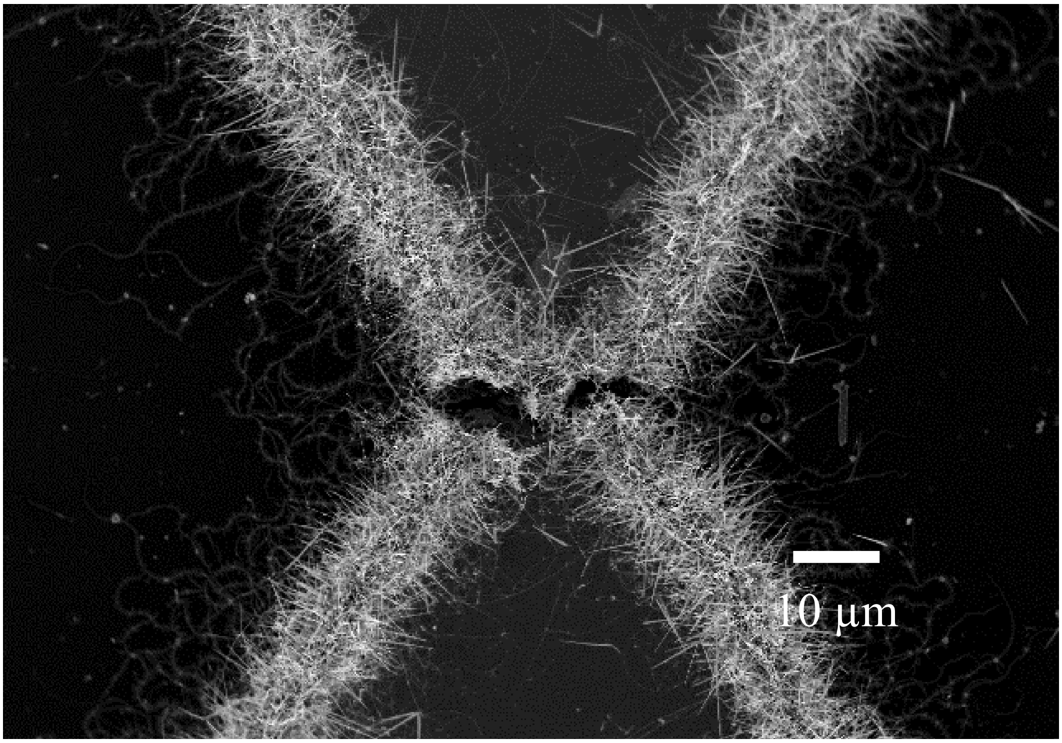

Figure S4. Stability of sensor S1 after a month continuous operation at supplied power of 10 μW.

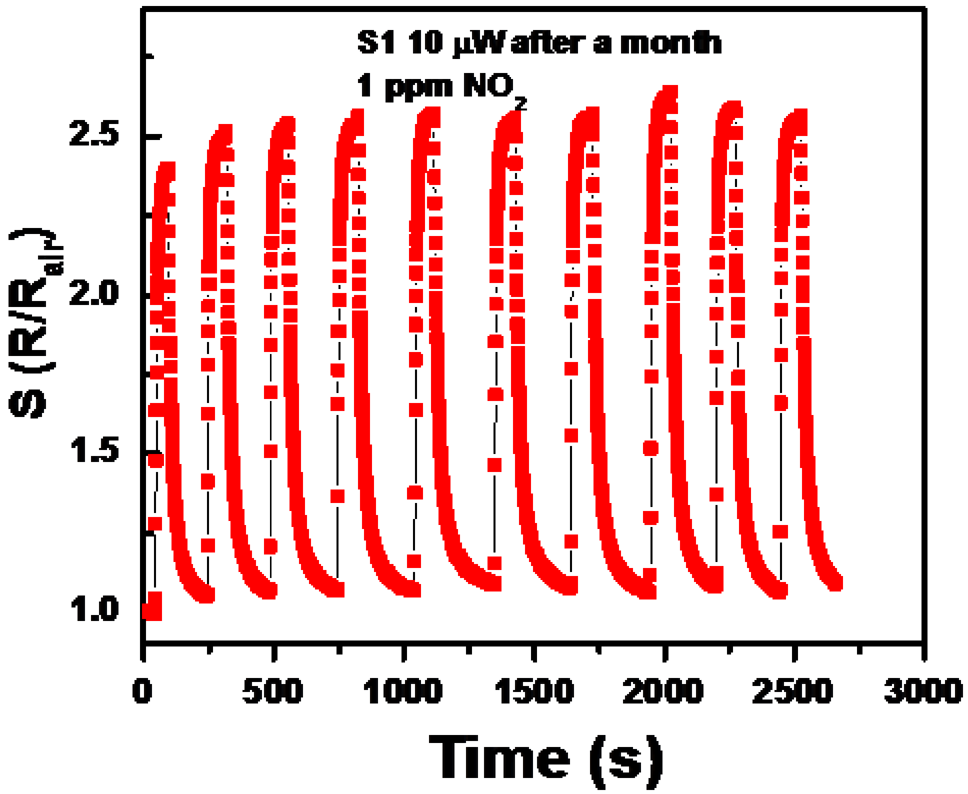

Supplement: RA-008-C8RA06061D-s001 [file RA-008-C8RA06061D-s001.pdf]
